# Short-term outcome of plasma adsorption therapy in amyotrophic lateral sclerosis

**DOI:** 10.5937/jomb0-40631

**Published:** 2023-08-25

**Authors:** Bin Li, Wei Zhang, Shaoxin Zhong, Jianyi Pan, Xiaohong Wang, Hequn Zou, Xianrui Dou

**Affiliations:** 1 Shunde Hospital of Southern Medical University, Department of Nephrology, Shunde, China; 2 The Third Affiliated Hospital of Southern Medical University, Department of Nephrology, Guangzhou, China; 3 South China Hospital Affiliated to Shenzhen University, Department of Nephrology, Shenzhen, China

**Keywords:** amyotrophic lateral sclerosis, plasma adsorption, therapeutic effect, amiotrofična lateralna skleroza, adsorpcija plazme, terapeutski efekat

## Abstract

**Background:**

To observe the short-term outcome of plasma adsorption PA therapy in amyotrophic lateral sclerosis (ALS).

**Methods:**

28 cases of als patients were recruited in this study, of which 20 were male and 8 were female with a mean age of 53.21±9.07 years and the average course of 33±23.35 months. The clinical manifestations were limb weakness (N=27), muscular atrophy (N=27), muscular tremor (N=5), dysphagia (N=12) and dysarthria (N=12). The clinical data of the patients recruited were graded by Amyotrophic Lateral Sclerosis Functional Rating Scale Revised (ALSFRSR) : <10 (N=1), 11-20 (N=4), 21-30 (N=6), 31-40 (N=12), >40 (N=5). All patients received PA therapy once a week for three successive times after examining the conditions of blood coagulation and virus infection. PA therapy was supplemented with neurotrophic therapy meanwhile. All patients' clinical manifestations and scores of ALSFRSR before treatment and one week after treatment were evaluated and compared. The levels of serum superoxide dismutase (SOD), interleukin-10 (IL-10), serum creatine kinase (CK) and lactate dehydrogenase (LDH) before and after treatment were compared.

**Results:**

After PA therapy, 14 patients have improved obviously in muscle strength, 4 patients in hypermyotonia partially, 3 patients in muscular tremor, 5 patients in dysarthria, 3 patients in salivation to some extent and 2 patients in swallowing function. The score of ALSFRSR after PA treatment (31.89±10.36) was remarkably higher than that before PA treatment (30.68±10.52) (P<0.01). The levels of SOD (155.10±21.87 IU/L) and IL-10 (138.06±185.88 pg/mL) after PA treatment were significantly higher than the levels before PA treatment (143.08.3±19.16 IU/L and 46.34±75.31 pg/mL, respectively) (P<0.05). The levels of CK (168.86±113.50 IU/L) and LDH (152.07±32.65 IU/L) after PA treatment were significantly lower than the levels before PA treatment (356.68±250.30 IU/L and 181.36±33.74 IU/L respectively) (P<0.01). At the end of follow-up period (November, 2019), five patients died of respiratory failure 16-21 months after PA treatment and two patents died of respiratory infection 15-20 months after PA treatment. 7 patients were still alive. The score of ALSFRS-R of these patients who survived at the end of follow-up (13.00±13.37) were significantly lower than before PA treatment (36.71±8.56) (P<0.05) and after PA treatment (38.14±8.82) (P<0.05).

**Conclusions:**

Plasma adsorption (PA) therapy has shortterm therapeutic effects on als. The effects might be attributed to the anti-oxygen free radical effect by increasing SOD level and the anti-inflammation effect by increasing IL-10 level. As the efficacy of PA therapy was obtained in a small sample size and short follow-up period, the longterm observation of PA efficacy in treating als should be further investigated.

## Abstract (sr)

**Uvod:**

Cilj je bio da se posmatra kratkoročni ishod terapije adsorpcije plazme (PA) kod amiotrofične lateralne skleroze (ALS).

**Metode:**

U ovoj studiji je regrutovano 28 slučajeva obolelih od als, od kojih su 20 bili muškarci i 8 žene sa prosečnom starošću od 53,21±9,07 godina i prosečnim tokom 33±23,35 meseci. Kliničke manifestacije bile su slabost udova (N=27), mišićna atrofija (N=27), mišićni tremor (N=5), disfagija (N=12) i dizartrija (N=12). Klinički podaci regrutovanih pacijenata su ocenjeni revidiranom funkcionalnom skalom amiotrofične lateralne skleroze (ALSFRSR): <10 (N=1), 11-20 (N=4), 21-30 (N=6), 31-40 (N=12), >40 (N=5). Svi pacijenti su primali PA terapiju jednom nedeljno tri puta uzastopno nakon ispitivanja stanja koagulacije krvi i virusne infekcije. PA terapija je u međuvremenu dopunjena neurotrofnom terapijom. Procenjene su i upoređene kliničke manifestacije svih pacijenata i rezultati ALSFRSR pre tretmana i nedelju dana nakon tretmana. Upoređeni su nivoi serumske superoksid dismutaze (SOD), interleukina-10 (IL-10), serumske kreatin kinaze (CK) i laktat dehidrogenaze (LDH) pre i posle tretmana.

**Rezultati:**

Nakon terapije PA, kod 14 pacijenata je došlo do očiglednog poboljšanja mišićne snage, 4 pacijenta u delimičnoj hipermiotoniji, 3 pacijenta u mišićnom tremoru, 5 pacijenata u dizartriji, 3 pacijenta u delimičnoj salivaciji i 2 pacijenta u funkciji gutanja. Skor ALSFRSR posle tretmana PA (31,89±10,36) bio je značajno viši od onog pre tretmana PA (30,68±10,52) (P 0,01). Nivoi SOD (155,10±21,87 IU/L) i IL-10 (138,06±185,88 pg/mL) nakon tretmana PA su bili značajno viši od nivoa pre tretmana PA (143,08,3±19,16 IU/L i 46,34±75.31 pg/mL, respektivno) (P 0,05). Nivoi CK (168,86±113,50 IU/L) i LDH (152,07±32,65 IU/L) nakon tretmana PA bili su značajno niži od nivoa pre tretmana PA (356,68±250,30 IU/L i 181,36±32,65 IU/L) respektivno. (P 0,01). Na kraju perioda praćenja (novembar 2019.), pet pacijenata je umrlo od respiratorne insuficijencije 16-21 mesec nakon tretmana PA, a dva pacijenta su umrla od respiratorne infekcije 15-20 meseci nakon tretmana PA. 7 pacijenata je još uvek bilo živo. Skor ALSFRS-R ovih pacijenata koji su preživeli na kraju praćenja (13,00±13,37) bio je značajno niži nego pre tretmana PA (36,71±8,56) (P<0,05) i posle PA tretmana (38,14±8,82) ( P<0,05).

**Zaključak:**

Terapija adsorpcijom plazme (PA) ima kratkoročne terapijske efekte na als. Efekti se mogu pripisati dejstvu slobodnih radikala protiv kiseonika povećanjem nivoa SOD i anti-inflamatornom efektu povećanjem nivoa IL-10. Kako je efikasnost PA terapije dobijena u maloj veličini uzorka i kratkom periodu praćenja, dugotrajno posmatranje efikasnosti PA u lečenju als-a treba dalje istražiti.

## Introduction

Amyotrophic lateral sclerosis, also known as gradually frozen people, is a kind of progressive degenerative disease violating spinal cord, brain stem and cortical motor neurons with unknown pathology. The average survival from onset to death is 3–5 years. The average age of onset of ALS patients is about 55 years with the incidence rate of 10/million-30/million and prevalence rate of 40/million-60/million, mainly in the middle-aged and the elderly people. It has the tendency of familial heredity (5–20% of patents). The etiology and pathogenesis of the disease are not fully understood and there is no effective way to stop the progression of the disease. Riluzole is approved by FDA in clinical treatment of the disease, which can extend the survival of patients to some extent, but no reversal of the disease [1].

PA is a new blood purification technology widely used in the treatment of rheumatic diseases and some neurological diseases, including myasthenia gravis, multiple sclerosis diseases and so on [2] [3]. The purpose of this study is to observe the efficacy of PA in the treatment of ALS and to explore its therapeutic mechanism.

## Materials and methods

### Objects

This study involved 28 patients (20 male and 8 female) diagnosed with ALS between September 2017 and December 2017 in the Department of the Third Affiliated Hospital of Southern Medical University. The average age of the patients was 53.21 ±9.07y (37–74y). The course of the disease was 33±23.35 months (10–108 m). All patients met the diagnostic criteria of EI Escorial ALS [4]. The clinical manifestations of these patients were: limbs weakness (N=27), muscular atrophy (N=27), muscular tremor (N=5), dysphagia (N=12) and dysarthria (N=12). After admission, all patients were scored according to the Amyotrophic Lateral Sclerosis Functional Rating Scale Revised (ALSFRS-R) [5] : <10 (N=1), 11–20 (N=4), 21–30 (N=6), 31–40 (N=12), >40 (N=5).

### Treatment

This study was approved by the Ethics Committee of the third Affiliated Hospital of Southern Medical University before practice. All participants gave their informed consent before treatment. The indicators of blood coagulation and virus infection of all patients were normal before PA treatment.

Plasauto-IQ plasma purification machine (Asahi Kasei Corporation, Japan), plasma separator OP-08W and adsorber IMMUSORBA TR-350 (Asahi Kasei Cor poration, Japan) were used in this study. Blood flow was 80–120 mL/min and the slurry velocity was 1.5 L/h. One and a half times of the Plasma volume of a patient was purified for each treatment. 20 mg dexamethasone and 25 mg phenergan were used to prevent anaphylaxis before treatment. PA treatment was conducted once a week in three consecutive weeks, while supplemented with neurotrophic therapy. 4 weeks after PA treatment the clinical manifestations, ALSFRS scores of the patients were recorded and the serum level of superoxide dismutase (SOD), interleukin-10 (IL-10), creatine kinase (CK) and lactate dehydrogenase (LDH) were tested.

### Statistical analysis

All analyses and calculations were performed by using SPSS (version 17.0). Measurement data were shown as mean ± standard deviation (SD). Paired t tests were used to compare the changes before and after treatment. A two-tailed P values <0.05 were considered statistically significant.

## Results

### Changes of clinical manifestations of ALS patients after PA treatment

14 patients have improved obviously in muscle strength, 4 patients in hypermyotonia partially, 3 patients in muscular tremor, 5 patients in dysarthria, 3 patients in salivation and 2 patients in swallowing function to some extent.

### Changes of ALSFRS-R of ALS patients after PA treatment

After treatment, the average ALSFRS-R score was significantly increased from 30.68±1.99 (before treatment) to 31.89±1.96 (after treatment) (P<0.05).

### Changes of serum SOD and IL-10 after PA treatment

As is shown in Table 1, serum SOD and IL-10 were significantly increased after PA treatment in ALS patients compared with those before treatment (P <0.05).

**Table 1:** Changes of Serum SOD and IL-10 after PA Treatment in ALS Patients. *: P <0.05

	SOD (IU/L)	IL-10 (pg/mL)
Before <br>Treatment	143.08.3±19.16	46.34±75.31
After <br>Treatment	155.10±21.87*	138.06±185.88*

### Changes of serum CK and LDH after PA treatment

As is shown in Table 2, serum CK and LDH were significantly decreased after PA treatment in ALS patients compared with those before treatment (P <0.05).

**Table 2:** Changes of Serum CK and LDH after PA Treatment in ALS Patients. ∆: P <0.01

	CK (IU/L)	LDH (IU/L)
Before <br>Treatment	356.68±250.30	181.36±33.74
After <br>Treatment	168.86±113.50∆	152.07±32.65∆

### Follow-up results of ALS after PA treatment

At the end of follow-up (November, 2019), five patients died of respiratory failure 16–21 months after PA treatment and two patents died of respiratory infection 15–20 months after PA treatment. 7 patients were still alive. The scores of ALSFRS-R of these patients who survived at the end of follow-up (13.00±13.37) were significantly lower than before PA treatment (36.71±8.56) (P<0.05) and after PA treatment (38.14±8.82) (P<0.05). The other patients were lost follow-up.

## Discussion

ALS is a major type of motor neuron disease, which selectively violates the anterior horn of spinal cord, motor neurons of brain stem, pyramidal cells of cortex and pyramidal tract. Clinically, the main manifestations are myasthenia, muscle atrophy, muscular tremor, hypermyotonia and tendon hyperreflexia. Sensory function and sphincter function are generally not affected. Its pathogenesis is not yet clear but there are several candidate theories including excitatory amino acid toxicity theory, gene mutation theory, oxidative stress theory [6] [7], autoimmune theory [8], etc. In excitatory amino acid toxicity theory, it is accepted that there is a transport disorder of high-affinity glutamic acid. Due to this disorder, the extra-cellular glutamic acid can not be eliminated and subsequently cause cell damages by increasing excitatory toxicity.

Oxidative stress theory refers to a pathological state that free radicals and their products generating from metabolism of tissues exceed the antioxidative defense of body leading to extensive oxidation of proteins, DNAs and lipids which cause damages of biological membranes, and nucleic acid of neurons. There were some evidences for oxidative damage of proteins, lipids and nucleic acids, both in ALS patients and cellular models [9] [10]. Studies have found that the concentration of DNA damage marker 8- hydroxy 2- acid, the content of malondialdehyde and other content of lipid peroxidation products were increased in ALS patients’ cerebrospinal fluid and cerebral cortex [11]. Based on the theory of oxidative stress, antioxidants and free radical scavenging agents have been used in the treatment of ALS, which yielded certain effects. Studies have shown that vitamin E, an antioxidant and free radical scavenger, could slow down the progression of the disease [12]. Nagase et al. [13] suggested that edaravone, as a free radical scavenger, can prevent the development of ALS to some extent.

In recent years, with the development of immunology and molecular biology, the role of immunological factors in the pathogenesis of ALS has been widely recognized. The immune mechanism of ALS is complex, including the deposition of immunoglobulin and immune complex in nerve tissues, anti-neuronal antibodies in blood and cellular immunity [8] [14]. Following studies supported immune theory including the findings of anti-GM1- IgM antibody and IgG antibody of L- type voltage depending 1 subunit of calcium channel in the blood of a small number of ALS patients as well as effectiveness of immunosuppressant in some clinical trails [15].

Large amounts of data indicate that the inflammatory reaction plays a certain role in the pathogenesis of ALS. Expression of inflammatory cytokines can be detected in the brain tissue of ALS patients such as interleukin -18, RIPK3 and NLRP3, etc [16].

There is no markedly effective therapy for this disease so far. The main treatment method depends on excitatory amino acid toxicity theory and oxidative stress theory. As for excitatory amino acid toxicity theory, the major medicines include glutamic acid suppressants and glutamic acid modulators with an aim of suppressing toxicity

Ruluzole, as the first medicine which successfully extended lifespan of ALS patients and the only medicine affirmed by FDA as an effective drug for ALS, can block excitatory amino acid toxicity by suppressing release of glutamic acid at presynaptic sites and interrupting the effect of excitatory amino acid at postsynaptic sites. It can extend the survival time for patients from 11.8 to 14.8 months [1], but still cannot reverse the progression of the disease. The main glutamic acid modulator is branched chain amino acids which can improve muscle strength and walking ability by activating glutamate dehydrogenase and further modulate metabolism or delivery of glutamate. As noted before that excitatory amino acid toxicity theory only suit for a minority of ASL patients, these therapies just make effect in some of the patients. Medicines, in Accordance with the oxidative stress theory, are vitamin E and C, acetyl cysteine, Edaravone, etc. Acetyl cysteine, serving as a scavenger of free radicals, is a precursor of glutathione which is the main antioxidant system. Other treatment methods include nerve nutrition therapy [17] and stem cell transplantation [18], are still in pre-clinical and early clinical research.

Plasmapheresis is group of new blood purification methods. By plasmapheresis, pathogenic materials can be eliminated throng a variety of measures after plasma was separated. The therapeutic range of plasmapheresis covers more than a hundred diseases. The main aim is to eliminate pathogenic materials, especially inflammatory factories and toxic materials as well as immune factors [19].

There are amount of reports about plasmapheresis in the field of inflammatory and rheumatic diseases. The strength and advantage of plasmapheresis is its rapid and effective elimination of pathogenic materials, and by this way interrupting inflammation cascade or immune cascade. Plasmapheresis may rapidly control disease condition. Pharmaco - therapy, if combined with plasmapheresis, may enhance its therapeutic effect and reduce side effects as well as delay relapse. Recent studies have shown that plasmapheresis may modulate immune system and regain cell immunologic functions and phagocytosis of reticuloendothelial cells [20].

Plasmapheresis include a variety of methods such as plasma exchange (PE), double filtration plasma pheresis (DFPP), Cryofiltration, Thermofiltration, Heparin extracorporal LDL precipition (HELP) and Plasma adsorption (PA).

PA therapy is a type of blood purifications, which is now used to remove pathogenic substances (pathogenic antibodies and inflammatory factors, etc) in plasma separated from blood by an adsorber. PA treatment is mainly used in renal diseases, rheumatic diseases and nervous system diseases such as systemic lupus erythematosus and lupus nephritis, myasthenia gravis and Guillain-Barre syndrome [3]. Different pathogenic substances can be adsorbed depending on the different adsorbers. With alanine as the ligand, immune complexes, anti DNA antibodies, rheumatoid factors and other pathogenic substances can be adsorbed. This adsorber can be used to treat diffuse connective tissue disease. With tryptophan as ligand, immune complexes, such as anti-acetylcholine receptor antibodies and other autoantibodies, can be removed by selectively hydrophobic binding and this is benefited to some nervous system diseases.

Due to the relatively specific elimination of pathogenic materials and no need of blood transfusion, the risk of blood transmitted diseases infection or anaphylactic reaction will be avoided as well as consumption of blood. Moreover, because there is no contact between absorbent and blood formed elements. The blood cells would not be destroyed by the treatment. Meanwhile, the efficacy of adsorption will be higher because of little interference factors in plasmapheresis. This kind of therapy represents the further direction of blood purification. In recent years, we have successively treated many inflammatory and immunological diseases such as myasthenia gravis, Guillain-Barre syndrome, multiple sclerosis, Chronic inflammatory demyelinating sheath of nerve root inflammation, severe lupus, severe dermatomyositis and severe rheumatoid arthritis. We also made some pilot studies in treating dilated cardiomyopathy, idiopathic pulmonary fibrosis, sclerosing cholangitis and autoimmune hepatitis. These diseases have similar inflammatory and autoimmune pathogenesis. We have got encouraging results in dilated cardiomyopathy and idiopathic pulmonary fibrosis. These results have paved the way for treating ALS which is believed to have pathogeneses of inflammatory and immunological reactions.

Our preliminary study showed that plasma adsorption has a certain efficacy in the treatment of rheumatic diseases and ALS. In this study, the therapeutic effects of PA in the 28 ALS patients have been observed and the possible therapeutic mechanism has been explored. This study shows that PA can improve patient’s clinical symptoms to some extent. After treatment, the ALSFRS-R score was 31.89±1.96, which was significantly increased compared with the score 30.68±1.99 (before treatment) (P<0.05). This research also indicates that after PA treatment the serum SOD and IL-10 have significantly increased than those before treatment (P<0.05), suggesting that PA treatment may reduce oxidative stress response and ameliorate the damage caused by oxidative stress and inflammatory reaction. Other studies have shown that anti-acetylcholine receptor antibody presents in the sera of ALS patients [14], and TR350 adsorption column can adsorb anti-acetylcholine receptor antibody, suggesting that PA treatment can achieve the goal of treatment through the elimination of anti-acetylcholine receptor antibodies and other pathogenic antibodies. In addition, this study finds that after PA treatment the serum CK and LDH have significantly decreased, indicating that PA may improve the inflammatory state of skeletal muscle in ALS patients.

In summary, this study shows that PA can improve the symptoms and signs of ALS patients in a short term, speculating its treatment mechanism may relate to that PA can inhibit patients’ immune response, eliminate patients’ autoantibodies and other inflammatory factors, reduce oxidative stress response and alleviate inflammation of skeletal muscle. Due to the small sample size and short follow-up period, this study may have some limitations. The long-term curative effects of plasma adsorption need to be further verified by large sample and long-term observation in the future.

## Dodatak

### Financial Disclosure

The authors declared that this study has received no financial support.

### Conflict of interest statement

All the authors declare that they have no conflict of interest in this work.
